# The relationship between the level of economic development, the scale of youth football development and competitive strength in Chinese provinces

**DOI:** 10.1371/journal.pone.0340359

**Published:** 2026-01-28

**Authors:** Peng Shi, Shunding Hu, Ziyun Zhang

**Affiliations:** 1 School of Physical Education, Shandong University of Technology, Zibo, Shandong, China; 2 School of Physical Education, Liaoning Normal University, Dalian, Liaoning, China; 3 School of Life and Health, Huzhou College, Huzhou, Zhejiang, China; Universiti Malaya, MALAYSIA

## Abstract

This study aims to explore the relationship between the economic development level of Chinese provinces, the scale of youth football development, and competitive strength. The data are derived from 31 provinces of China (excluding Hong Kong, Macao, and Taiwan). Indicators of economic development level include GDP, per capita GDP, per capita disposable income, and per capita consumption expenditure. Indicators of the scale of youth football development include the number of featured schools, the number of campus football fields, the level of football clubs in the three-tier league system, the number of social brand youth training institutions, and the number of sports schools. Indicators of youth football competitive strength include the number of top teams and elite teams in the Chinese Youth Football League. The study found that after controlling for total population size and youth population size, per capita disposable income showed a marginally significant positive correlation with both the number of top teams (*r* = 0.320, *P* = 0.091) and the number of elite teams (*r* = 0.359, *P* = 0.056); GDP (*r* = 0.458, *P* = 0.012), per capita GDP (*r* = 0.466, *P* = 0.011), per capita disposable income (*r* = 0.414, *P* = 0.026), and per capita consumption expenditure (*r* = 0.412, *P* = 0.026) all exhibited a moderately significant positive correlation with the number of social youth training institutions; per capita disposable income (*r* = −0.469, *P* = 0.010) and per capita consumption expenditure (*r* = −0.448, *P* = 0.015) showed a moderately significant negative correlation with the number of featured schools; the level of clubs in the three-tier league system displayed a highly significant positive correlation with both the number of top teams (*r* = 0.648, *P* < 0.001) and the number of elite teams (*r* = 0.778, *P* < 0.001); and the number of featured schools had a weakly significant negative correlation with the number of top teams (*r* = −0.369, *P* = 0.049). In addition, the level of clubs in the three-tier league system played a mediating role between economic indicators and competitive strength. The above research findings can provide a scientific basis for the Chinese government to optimize football policy orientations and the allocation of financial resources.

## 1. Introduction

Youth football is a fundamental project for expanding the football population and identifying and cultivating professional talents [1]. Only by solidifying the foundation of youth football can we address the shortage of football talents and provide core support for the revitalization of Chinese football and the building of a strong sports nation [2]. In addition, using youth football as a key driver can promote the optimization of physical education teaching and the improvement of facilities in schools, laying a solid foundation for the national fitness campaign and the Healthy China strategy [3,4]. The development of youth football can drive the growth of the football industry and generate substantial economic value [5]. Therefore, youth football occupies a core position in the football system, and the reform and development of Chinese football have always been carried out with youth football at its center. The Chinese government has issued a number of policy documents to guide the development of youth football. Up to now, a diversified training system composed of youth sports schools, campus football programs, and social institutions has gradually taken shape [6].

In China, provincial government departments or agencies are the key levels for policy implementation [7], the focal points for the promotion of youth football-related policies in China, and the critical levels for the selection of members of the national youth and junior teams. Therefore, the competitive development of provincial youth football is a segment that Chinese government departments focus on with great attention. With the rapid development of China’s economy, there are significant differences in economic volume, development speed, and industrial structure among provinces. This imbalance in provincial economic development is related to the allocation of sports resources and infrastructure construction [8]. Therefore, the scale of youth football development and football competitiveness in a local area largely depend on the local level of economic development. Generally speaking, economically developed provinces are usually able to invest more funds in the structural development of youth football, increase the base number of youth football participants, and enhance the quality of elite youth football players, thereby improving the local football competitiveness.

In fact, existing studies have explored the correlation between economic development level and football competitiveness (see the literature review section for details), but there are some limitations. Specifically, the conclusions of previous studies are controversial, and there is a lack of systematic evaluation of the scalable elements in youth football development, such as clubs, campus football characteristic schools, social branded youth training institutions, and sports schools. In addition, there is also a lack of empirical research based on Chinese samples. Based on this, the present study, using data from Chinese provinces, explores the relationship between the economic development level of Chinese provinces and competitive strength, analyzes the scale layout of youth football at the provincial level in China, and discusses the mediating role of this scale layout in the relationship between the two. Theoretically, this study integrates the theories of regional competitive development and youth football development, enriching the theoretical research on how regional economies influence specific sub-sectors of the sports industry. It also provides a reference for further exploring the relationship between regional economies and the development of other sports. In addition, by analyzing the relationship between economic development and the scale and competitive strength of youth football, this study fills some gaps in the research on the mechanisms through which economic factors impact youth football development. Practically, the findings of this study can provide direct and scientific evidence for Chinese government departments to formulate policies on youth football development. It also offers data support for promoting the coordinated development of the football industry and regional economies.

## 2. Literature review

### 2.1. The correlation between socio-economic development, the scale of youth football development, and football competitive strength

Currently, relevant studies have explored the correlation between indicators related to the level of socio-economic development and football competitive strength. Mourão [9] found that per capita income is an important factor for the emergence of new first-division league teams in Portuguese cities. Zahid [10] investigated the determinants of success for football teams in the African Football Association and discovered the positive role of wealth in football performance. In addition, Klobučník et al. [11] explored the relationship between the sporting performance of football clubs and the GDP of their respective regions in the European Union, finding a moderate positive correlation between regional GDP and sporting performance. Meanwhile, some special phenomena were observed: clubs in certain regions with relatively low economic levels (such as Northern Portugal and the Community of Madrid, Spain) achieved outstanding sporting results, while clubs in some high-GDP regions (such as Île-de-France, France) underperformed expectations.

However, there are studies that do not support the aforementioned positive correlation. Fan et al. [12] collected panel data from 68 countries (regions) participating in the World Cup finals from 1994 to 2022 for analysis and found that there is a significant negative correlation between the Human Development Index of countries (regions) and their World Cup performance, while the comprehensive benefit of a country’s (region’s) economic development level and football tradition on World Cup results is positive and significant. Vorobyev et al. [13] found that the correlation between the official FIFA men’s football ranking and the level of socio-economic development is not significant. Hoffmann et al. [14] found an inverted U-shaped relationship between per capita wealth and national team performance in international competitions. In addition, there are already studies that have confirmed the correlation between the economic development level of Chinese provinces and the scale of youth football development. Yun et al. [15] found that there is a significant positive correlation between provincial GDP and the number of campus football characteristic schools, and that government funding is highly positively correlated with football field facilities. Zhou and Tang [16] discovered that the development of professional football clubs is associated with the regional economic level. In summary, although the field has accumulated a considerable amount of research, the results are inconsistent, which may be related to the inconsistency in the selection of indicators and the differences in culture among countries (regions).

Current studies have the following limitations. Firstly, as can be seen from the above review, the results of previous studies are inconsistent. The relationship between the level of provincial economic development, the scale of youth football development, and the competitive strength of youth football is found to be positive, negative, or in the form of an inverse U-shaped relationship. Secondly, youth football is a systematic project [17], and a comprehensive assessment of the relationship between provincial economic development levels and the scale of youth football is still needed, but there is a relative lack of comprehensive studies on the three major training systems (characteristic schools, social institutions, and sports schools). Thirdly, although previous studies [9,10,12–14] have explored the correlation between economic development levels and football competitive strength, there is a lack of research with Chinese samples, and a lack of studies with Chinese provincial samples, so it is not clear about the correlation between economic development levels and football competitive strength from a provincial perspective. Lastly, logically, the economic development level of a province can promote the development scale of youth football, and thus promote its football competitiveness, but there is a relative lack of empirical research at present.

### 2.2. The theoretical basis related to this study

#### 2.2.1. Resource-based view.

The Resource-Based View (RBV) is a classic theory in the field of strategic management, whose core proposition is that an organization’s competitive advantage stems from its heterogeneous, scarce, inimitable, and non-substitutable resources and capabilities [18]. These resources include both tangible resources (such as capital, infrastructure, equipment, etc.) and intangible resources (such as talent, technology, brand, organizational capabilities, etc.) [19]. By identifying, accumulating, and allocating these unique resources, organizations can build sustainable competitive advantages, thereby improving performance in the market or industry [19]. This study adopts the RBV as the core framework to analyze the relationship between provincial economic development and youth football development. Firstly, economic resources are the core tangible resources for youth football development. Secondly, per capita income and consumption capacity are important carriers of implicit resources.

#### 2.2.2. The systems theory of youth football development.

The systems theory originates from General Systems Theory, whose core proposition is that everything is an organic system composed of interrelated and interacting elements, and the overall function of the system depends on the synergistic relationship between elements rather than the isolated role of a single element [20,21]. Elements within the system (such as subjects, resources, environment, etc.) form a dynamic balance through the exchange of materials, energy, or information, and changes in any element will affect other elements and the overall function of the system through a transmission effect [20,21]. Meanwhile, the system also maintains continuous interaction with the external environment (such as economy, policy, culture). This study regards the development of youth football as an organic system with multi-element synergy, and clearly identifies the core elements of the youth football development system, including the input element of socio-economic development level, the mediating element of youth football development scale, and the output element of youth football competitive strength. The three form an indispensable chain structure of “resource input → scale transmission → strength output”.

## 3. Methods

### 3.1. Data sources

The research area of this study encompasses 31 provinces, municipalities, and autonomous regions of our country (excluding Hong Kong, Macao, and Taiwan). The collected data mainly includes three categories: the economic development level, the scale of youth football development, and the competitive strength of youth football in each province. The collection of all data is up to June 2024.

### 3.2. Variables

#### 3.2.1. Economic development level of provinces.

The economic development level of provinces is the independent variable in this study. Referring to previous studies [15,22–24], this study selects four indicators for 2023: GDP, per capita GDP, per capita disposable income, and per capita consumption expenditure to measure the economic development level of provinces. In addition, the rationale for selecting the aforementioned economic indicators is that they are logically associated with the scale of youth football development and the competitive strength of youth football. A higher provincial GDP signifies a stronger overall economic capacity, which implies more financial resources can be allocated to the development of youth football. A higher per capita GDP indicates that residents have more resources and willingness to invest in the cultivation of youth football. Moreover, disposable income per capita and per capita consumption expenditure directly determines a family’s ability to invest in youth football. Families with higher incomes are more capable of covering the costs associated with football training, competitions, and equipment for their children, thereby promoting the expansion of the scale of youth football development.

The definitions of the above indicators of provincial economic development levels are as follows. The GDP of a province refers to the final outcome of all production activities by all resident units in a province over a certain period; per capita GDP refers to the GDP of a province divided by the population of that province. Disposable income for residents refers to the total amount of income available for final consumption expenditure and savings, that is, the income available for free disposal, and per capita disposable income for each province refers to the disposable income of residents in each province divided by the number of permanent residents. Resident consumption expenditure refers to all expenditures made by residents to meet the daily living consumption needs of the family, including both cash and in-kind consumption expenditures, and per capita consumption expenditure for each province refers to the consumption expenditure of residents in each province divided by the number of permanent residents.

The relevant data comes from the China Statistical Yearbook 2024, which can be accessed at https://www.stats.gov.cn/sj/ndsj/2024/indexch.htm. Documents published in 2024 contain relevant data from 2023. The China Statistical Yearbook is a reference annual published by the National Bureau of Statistics of China, which comprehensively reflects the economic and social development of China. A given year’s statistical yearbook includes a vast array of statistical data from the previous year on various aspects of the economy and society at the national and provincial levels, making it the most comprehensive and authoritative comprehensive statistical yearbook in China.

#### 3.2.2. Scale of youth football development.

Currently, the youth football training system in China mainly consists of three types of entities: campus football featured schools, social organizations, and sports schools [3,4]. Therefore, this study primarily selects representative indicators that reflect the scale of provincial youth football development from the aforementioned three systems.

Featured schools for campus football are the main battleground for youth football, and the development level of campus football is related to the quantity and quality of the football population [25]. Since its development from 2015 to the present, relevant policies have continuously promoted the scale layout of featured campus football schools, as well as the construction of supporting football field facilities through renovation, transformation, new construction, and expansion. Based on this, this study uses the number of featured campus football schools and campus football fields in each province to represent the scale layout of the campus football training system. The featured campus football schools are sourced from the relevant lists published by the Office of the Ministry of Education; including data from 2015 to 2023 (data for 2022 is missing). In 2022, the Office of the Ministry of Education did not release the relevant list, so data from that year are not considered. This study sums up the number of featured campus football schools approved in each province over the eight years to obtain the number of featured campus football schools in each province. In addition, we reviewed government report [24] and excluded a total of eight schools that had their campus football program qualifications revoked. The number of campus football fields in each province comes from the latest statistical data published by the Ministry of Education in 2022 (http://www.moe.gov.cn/jyb_sjzl/moe_560/2022/).

In the era of professionalization and marketization, social youth training institutions play a significant role in China’s football youth training system. Professional clubs not only play an important role in training and selecting elite players, providing professional training and competition opportunities, but also promote the overall development of football by stimulating social enthusiasm and cultural atmosphere for the sport. Social brand youth training institutions typically have professional coaching teams and advanced training facilities, which can provide high-quality football education and training [26,27]. Therefore, this study uses the number of football clubs in the third-tier league and social youth training institutions to measure the scale of the social institution-based training system in each province. The data on the three-tier league clubs come from the official websites of the Chinese Football Association for the 2023 professional leagues, including the Super League, China League One, and China League Two clubs (http://www.csl-china.com/). This study assigns weights to the three-tier leagues based on their social popularity. By retrieving the daily average comprehensive indices of “Chinese Super League”, “China League One” and “China League Two” on the PC and mobile terminals in 2023 through Baidu Index (link: https://index.baidu.com/v2/index.html#/), the indices are 25,969, 3,159 and 583 respectively. After calculation, the weights of Chinese Super League, China League One and China League Two are assigned as 44.54, 5.42 and 1. Finally, the level of clubs in the three-tier leagues of each province is calculated by combining the event level and weight of clubs in each province. In addition, this study searched for “football clubs” on the Qichacha website (link: https://www.qcc.com/), set the search time frame to more than 3 years, and obtained the clubs established in each province before 2023.

Although sports schools face “stubborn diseases” such as narrow “import and export” channels, prominent contradictions between study and training, it cannot be denied that they are still an important part of China’s football youth training. For instance, studies [28,29] found that 22.0% of Chinese Super League players come from amateur sports schools, and 26.0% of players are registered with amateur sports schools. Therefore, this study uses the number of sports schools in each province that have been selected as national sports reserve talent bases (excluding single-sport schools that are not football) to represent the scale of the sports school training system in each province. The relevant data comes from the National Sports Reserve Talent Bases selected by the Youth and Sports Department of the General Administration of Sport in 2023.

#### 3.2.3. Competitive strength in youth football.

This study uses the number of teams from each province that advanced to the final and knockout stages in the 2024 China Youth Football League (CYFL) as effectiveness data. The CYFL is the top youth football event in China with the widest coverage, the largest number of participants, the highest level of competition, and the greatest social influence. It balances popularization and improvement, breaks down barriers to participation, and allows teams from all training systems to compete freely without any restrictions. The aim is to discover and select outstanding “talented young players” for intensive training and to provide talent for national teams at various levels [30]. Therefore, the CYFL data from each province directly reflects the competitive level of youth football in that province. Based on this, the study summarizes the number of teams from each province in the U13, U15, U17, and U19 final stages in 2024, and the number of top teams is obtained by summing up the teams of different age groups in each province. In addition, this study also aggregated the number of teams from each province that advanced to the national knockout stage across different age groups, to represent the number of elite teams in each province. The data were sourced from the official Weibo account of the Football Association of China. The data sources for the variables are detailed in Table 1.

**Table 1:** The data sources for the variables.

Variable Category	Specific Variable	Data Source
Economic Development Level of Provinces	1. GDP 2. Per capita GDP 3. Per capita disposable income 4. Per capita consumption expenditure	China Statistical Yearbook 2024 (accessible at https://www.stats.gov.cn/sj/ndsj/2024/indexch.htm)
Scale of Youth Football Development	1. Number of featured campus football schools (sum of approved schools in each province from 2015 to 2023, excluding 8 schools with revoked qualifications)	Relevant lists published by the Office of the Ministry of Education (2015–2023 data, 2022 data missing as no list was released that year); Government report [24] (for excluding schools with revoked qualifications)
	2. Number of campus football fields	The latest statistical data published by the Ministry of Education in 2022 (http://www.moe.gov.cn/jyb_sjzl/ moe_560/2022/)
	3. Level of clubs in the three-tier leagues (Super League, China League One, China League Two) in each province (calculated by combining event level and weight)	Official websites of the Chinese Football Association for the 2023 professional leagues (http://www.csl-china.com/) (for club distribution); Baidu Index (https://index.baidu.com/v2/index.html#/) (for obtaining 2023 daily average comprehensive indices to determine league weights: 44.54 for Super League, 5.42 for China League One, 1 for China League Two)
	4. Number of social youth training institutions (football clubs established before 2023 with operation period over 3 years)	Qichacha website (https://www.qcc.com/) (search keyword: “football clubs”, search time frame: established before 2023 with operation period over 3 years)
	5. Number of sports schools selected as national sports reserve talent bases (excluding non-football single-sport schools)	National Sports Reserve Talent Bases selected by the Youth and Sports Department of the General Administration of Sport in 2023
Competitive Strength in Youth Football	1. Number of top teams (sum of teams from each province in U13, U15, U17, U19 final stages of 2024 CYFL)	Official Weibo account of the Football Association of China (data on 2023 CYFL final stage participants)
	2. Number of elite teams (sum of teams from each province advancing to national knockout stage of 2024 CYFL across different age groups)	Official Weibo account of the Football Association of China (data on 2023 CYFL national knockout stage participants)

#### 3.2.4. Control variable.

To eliminate the interference of population size on the correlation analysis between provincial economic indicators and the development scale of youth football, this study selects the provincial population size and youth population size as control variables. The data on population size is sourced from the 2024 China Statistical Yearbook, where the 2024 data is mainly compiled based on the survey conducted in 2023. The data on youth population size comes from the 2023 education statistics released by the Ministry of Education of the People’s Republic of China. This dataset provides detailed statistics on the number of students receiving primary education, junior secondary education, secondary vocational education, and senior secondary education in each province of China. In this study, the youth population size of each province is obtained by summing up the aforementioned student numbers.

### 3.3. Data analysis

This study uses SPSS 21.0 and AMOS 12.0 software for data processing and statistical analysis. Specifically, SPSS 21.0 is mainly responsible for conducting descriptive statistics and correlation analysis, while AMOS 12.0 is mainly used for path analysis. Firstly, descriptive statistics were performed on variables using mean (*M*), standard deviation (*SD*), maximum (*Max*), and minimum (*Min*) values. Secondly, in this study, we used the one-sample Kolmogorov-Smirnov test to assess the normality of the data. The results indicated that the data did not follow a normal distribution. Therefore, this study first conducts a logarithmic transformation of the variables to make them approximately follow a normal distribution. After the logarithmic transformation, partial correlation analysis is adopted to explore the correlations between provincial-level economic development level, youth football development scale, and youth football competitive strength, with population size and youth population size controlled. This study, referring to previous studies [31,32], defines strong correlation as a correlation coefficient above 0.70, moderate correlation as a correlation coefficient between 0.40 and 0.69, and weak correlation as a correlation coefficient below 0.40. Finally, AMOS 12.0 software was used to construct a structural equation model. In this model, the logarithm of provincial-level economic development level served as the independent variable, the logarithm of provincial-level youth football development scale as the mediating variable, and the logarithm of youth football competitive strength as the dependent variable. The significance of the relationships among the three variables was evaluated by testing the path coefficients between them—if the path coefficients of both “independent variable → mediating variable” and “mediating variable → dependent variable” are significant, it indicates the existence of a mediating effect. In path analysis, this study used the 95% bias-corrected confidence interval generated by Bootstrap resampling to determine the significance of paths. In addition, the chi-square to degrees of freedom ratio (χ²/df), root mean square error of approximation (RMSEA), comparative fit index (CFI), standardized root mean square residual (SRMR), and non-normed fit index (NNFI/TLI) were employed to conduct the model fit analysis. The significance level for all statistical methods mentioned above was defined as *α* = 0.05. All data generated and analyzed during this study are included in the supplementary file (S1 File).

## 4. Results

### 4.1. Basic information about variables

The economic development level, scale of youth football development, and competitive strength in football for various provinces in China are detailed in Table 2. In terms of economic development level, the average GDP for each province is 40,348.45 billion yuan, the average per capita GDP is 88,203.68 yuan, the average per capita disposable income is 38,771.84 yuan, and the average per capita consumption expenditure is 26,336.03 yuan. In terms of the scale of youth football development, the average number of featured schools is 1,242.90, the average number of campus football fields is 4,555.68, the level of football clubs in the fourth-tier league system is 26.30, the average number of social football youth training institutions is 341.13, and the average number of sports schools is 18.48. In terms of youth football competitiveness, the average number of top teams is 7.65; the average number of elite teams is 3.10. In addition, in terms of controlling variables, the average population size is 45.2619 million, and the average youth population size is 6.4201 million.

**Table 2:** Basic information about variables.

Variable categories	Variables	*M*	*SD*	*Max*	*Min*
Economic development level	GDP	40348.45	32813.80	135673.00	2393.00
	Per capita GDP	88203.68	38000.42	200278.00	47867.00
	Per capita disposable income	38771.84	14698.03	84834.00	25011.00
	Per capita consumption expenditure	26336.03	8509.36	52508.00	17220.00
Scale of youth football development	Number of featured schools	1242.90	771.24	3162.00	145.00
	Number of campus football fields	4555.68	3159.20	13143.00	664.00
	Level of football clubs in the three-tier league system	26.30	33.09	102.92	0.00
	Number of social football youth training institutions	341.13	243.77	1093.00	14.00
	Number of sports schools	18.48	11.24	53.00	3.00
Competitive strength in youth football	Number of top teams	7.65	6.22	24.00	0.00
	Number of elite teams	3.10	3.66	13.00	0.00
Control variable	Population size	4526.19	3052.97	12706.00	365.00
	Youth population size	642.01	487.51	1850.22	63.82

### 4.2. The correlation between economic development level, scale of youth football development, and competitive strength in youth football

#### 4.2.1. The correlation between economic development level and competitive strength of youth football.

After controlling for population size and youth population size, the results of the correlation analysis between the economic development level of provinces and the competitive strength of youth football (Table 3) show that the indicators of provincial economic development level are positively correlated with those of youth football competitive strength. Specifically, the correlations between per capita disposable income and the number of top-tier teams (*r* = 0.320, *P* = 0.091) as well as the number of elite teams (*r* = 0.359, *P* = 0.056) have statistical significance at the marginal significance level; while the correlations between GDP, per capita GDP, per capita consumption expenditure and youth football competitive strength are all not significant (*P* > 0.05).

**Table 3:** Correlation between the economic development level of provinces and the competitive strength of youth football.

Variables	GDP	Per capita GDP	Per capita disposable income	Per capita consumption expenditure
Number of top teams	0.247	0.242	0.320#	0.258
Number of elite teams	0.221	0.215	0.359#	0.304

Note: # 0.1 < *P* < 0.05.

#### 4.2.2. The correlation between economic development level and scale of youth football development.

After controlling for the total population size and youth population size, the results of the correlation analysis between the provincial-level economic development level and the scale of youth football development (as shown in Table 4) revealed a certain degree of association between these two factors. Specifically, the gross domestic product (GDP) exhibited a moderately significant positive correlation with the number of social youth training institutions (r = 0.458, P = 0.012); the per capita GDP also showed a moderately significant positive correlation with the number of social youth training institutions (r = 0.466, P = 0.011); the per capita disposable income had a moderately significant negative correlation with the number of featured schools (r = −0.469, P = 0.010) while demonstrating a moderately significant positive correlation with the number of social youth training institutions (r = 0.414, P = 0.026); the per capita consumption expenditure displayed a moderately significant negative correlation with the number of featured schools (r = −0.448, P = 0.015) and a moderately significant positive correlation with the number of social youth training institutions (r = 0.412, P = 0.026). Notably, none of the indicators related to the provincial-level socioeconomic development level showed a significant correlation with the number of campus football fields, the level of clubs in the three-tier league system, or the number of sports schools (all P > 0.05).

**Table 4:** Correlation between the economic development level of provinces and scale of youth football development.

Variables	GDP	Per capita GDP	Per capita disposable income	Per capita consumption expenditure
Number of featured schools	−0.304	−0.305	−0.469*	−0.448*
Number of campus football fields	−0.080	−0.083	−0.185	−0.249
Level of football clubs in the three-tier league system	0.138	0.131	0.304	0.257
Number of social football youth training institutions	0.458*	0.466*	0.414*	0.412*
Number of sports schools	−0.014	−0.022	−0.028	−0.020

Note: *P < 0.05.

#### 4.2.3. The correlation between scale of youth football development and competitive strength in youth football.

After controlling for the total population size and youth population size, the results of the correlation analysis between the scale of youth football development and the competitive strength of youth football (Table 5) reveal that there is a certain correlation between the scale of provincial youth football development and the competitive strength of youth football. Specifically, the number of characteristic schools exhibits a low-degree significant negative correlation with the number of top-tier teams (r = −0.369, P = 0.049); the level of clubs in the three-tier league shows a high-degree significant positive correlation with both the number of top-tier teams (r = 0.648, P = 0.000) and the number of elite teams (r = 0.778, P = 0.000). In addition, it should be noted that the number of campus football fields, the number of social youth training institutions, and the number of sports schools have no significant correlation with the competitive strength of youth football (P > 0.05).

**Table 5:** Correlation between the scale of youth football development of provinces and competitive strength in youth football.

Variables	Number of featured schools	Number of campus football fields	Level of football clubs in the three-tier league system	Number of social football brand youth training institutions	Number of sports schools
Number of top teams	−0.369*	0.072	0.648**	0.229	0.014
Number of elite teams	−0.244	0.161	0.778**	0.106	0.200

Notes: *P < 0.05; **P < 0.01.

### 4.3. Test of the mediating role of the scale of youth soccer development between the economic development level and the competitive strength in football

**Fig 1:**
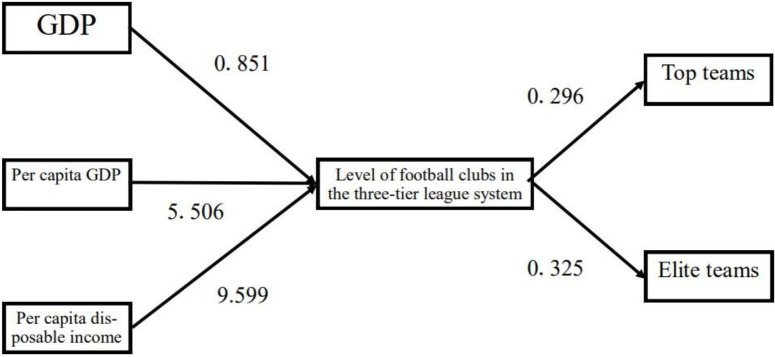
The mediating role of the scale of youth football development between provincial economic development levels and football competitive strength.

The results of the SEM fit test show that χ²/df = 2.504, indicating the model fit are within the acceptable range. Additionally, RMSEA = 0.224, CFI = 0.962, SRMR = 0.006, and TLI = 0.859. Overall, the model fit falls within the acceptable range. After controlling for total population size and youth population size, this study summarized the statistically significant paths, and the results (Table 6) showed that the paths from GDP to the number of featured schools (β = 0.906, P < 0.001), the number of campus football fields (β = 0.907, P < 0.001), the level of clubs in the three-tier league system (β = 0.851, P = 0.004), the number of social football youth training institutions (β = 0.867, P < 0.001), and the number of sports schools (β = 0.596, P < 0.001) were all significant; the paths from per capita GDP (β = 5.506, P = 0.003) and per capita disposable income (β = 9.599, P = 0.024) to the level of football clubs in the three-tier league system were significant; the path from per capita consumption expenditure to the number of campus football fields was significant (β = −1.654, P = 0.040); the path from the number of featured schools to the number of top teams was significant (β = −1.062, P = 0.012); and the paths from the level of clubs in the three-tier league system to the number of top teams (β = 0.296, P < 0.001) and the number of elite teams (β = 0.325, P < 0.001) were significant. In summary, the level of football clubs in the three-tier league system plays a significant mediating role between GDP, per capita GDP, per capita disposable income and the number of top teams, as well as between GDP, per capita GDP, per capita disposable income and the number of elite teams, with details of the mediating effect test shown in Fig 1.

**Table 6:** Path test results.

Path	*β*	*SE*	*CR*	*P*
Number of featured schools←GDP	0.906	0.053	17.156	<0.001
Number of campus football fields←GDP	0.907	0.055	16.489	<0.001
Level of football clubs in the three-tier league system←GDP	0.851	0.297	2.865	0.004
Number of social football youth training institutions←GDP	0.867	0.085	10.228	<0.001
Number of sports schools←GDP	0.596	0.090	6.586	<0.001
Level of football clubs in the three-tier league system←Per capita GDP	5.506	1.829	3.010	0.003
Level of football clubs in the three-tier league system←Per capita disposable income	9.599	4.264	2.251	0.024
Number of campus football fields←Per capita consumption expenditure	−1.654	0.804	−2.058	0.040
Number of top teams←Number of featured schools	−1.062	0.424	−2.505	0.012
Number of top teams←Level of football clubs in the three-tier league system	0.296	0.075	3.928	<0.001
Number of elite teams←Level of football clubs in the three-tier league system	0.325	0.061	5.336	<0.001

## 5. Discussion

This study surveyed data on provincial economic development levels, the scale of youth football development, and the competitive strength of youth football in China. It was found that there are varying degrees of correlation between provincial economic development levels and the number of provincial youth elite teams and top teams. Specifically, per capita disposable income has a marginal positive correlation with the number of top-tier teams and elite teams. The correlation between provincial economic development level and the scale of youth football development shows a differentiated characteristic: the significantly positive correlations are concentrated in social training institutions, the significantly negative correlation is reflected in featured schools, and some scale indicators have no correlation with economic level. The level of clubs in the three-tier league system has a positive correlation with the number of top-tier teams and elite teams, while the number of featured schools has a significant negative correlation with the number of top-tier teams. In addition, the level of clubs in the three-tier league system is the core bridge for the economy to affect competitive strength; that is, GDP, per capita GDP, and per capita disposable income positively affect the level of clubs in the three-tier league system, which in turn positively affects the number of top-tier teams and elite teams.

### 5.1. Per capita disposable income has a marginal positive correlation with the competitive strength of youth football

According to the perspective of the Resource-Based View (RBV) theory [33], the competitive advantage of enterprises or organizations stems from their unique resources and capabilities, which include tangible resources such as capital and intangible resources such as talent. This theory is applicable to the research topic of this study, that is, the level of economic development of each province determines the resources available for the development of youth football in that region. Among them, economically developed provinces can invest more funds in the development of youth football, thereby enhancing their competitive strength. The results are consistent with those of previous studies [10,12].

However, there are differences in the correlation between provincial economic development indicators and football competitive strength. The core reason may lie in the different orientations of various economic indicators. Among them, per capita disposable income is directly related to the ability of families to make independent investments in the field of football. In contrast, GDP, per capita GDP, and per capita consumption expenditure have a broader coverage, and their correlation with football competitive strength is more likely to be diluted by other factors.

Firstly, per capita disposable income reflects the funds that a household can freely dispose of, which can be directly converted into the “essential costs” for teenagers’ participation in football [34]. These costs include expenses such as paying for professional training camp fees, purchasing equipment, and covering transportation and accommodation costs for participating in inter-regional competitions. When the household disposable income reaches a certain level, parents are more likely to support their children in participating in long-term football training, rather than only focusing on short-term academic performance. This long-term investment is crucial for cultivating high-level competitive talents, as it can increase the number of top-tier and elite football teams in the region, which in turn accumulates into the overall strength of the region [35]. Secondly, GDP and per capita GDP measure the total economic output and average output of a region, covering multiple fields such as industry, agriculture, and services [36]. However, the proportion of resources directly invested in youth football—such as government special funds and corporate sponsorships for youth football training—may be extremely low, making it difficult to effectively drive the improvement of competitive strength. For example, the GDP growth of some regions may rely on resource-based industries or heavy industry; such an economic structure has weak relevance to the construction of a youth football training system and cannot be directly converted into football competitive strength. Finally, per capita consumption expenditure covers various aspects such as food, clothing, housing, transportation, medical care, and education [37,38]. The consumption spent on football, such as training and equipment, accounts for only a very small part. Moreover, it is easily affected by household consumption preferences—for instance, some households prefer art training over sports training—so it cannot be stably linked to football competitive strength. In addition, even if the per capita consumption expenditure in a region is high, if the region lacks high-quality youth football training resources (such as professional coaches and venues), the consumption demand cannot be converted into the actual improvement of competitive level, resulting in an insignificant correlation.

### 5.2. Correlation between provincial economic development level and the development scale of youth football shows a differentiated characteristic

The results of this study reveal that the correlation between the level of provincial economic development and the scale of youth football development presents a differentiated characteristic. Specifically, the positive and significant correlations of relevant economic development indicators are concentrated in social training institutions, the negative and significant correlations are reflected in featured schools, and some scale indicators show no correlation with the economic level. The core reasons for this can be explained from aspects such as the attribute differences of various development entities, resource dependence logic, and functional positioning.

Firstly, there is a positive correlation between provincial economic development indicators and social training institutions. Social youth football training institutions are market-oriented entities for youth football development, and their survival and expansion are highly dependent on residents’ consumption capacity, social capital investment, and market demand, which forms a direct and positive correlation with economic development indicators [39,40]. The operation of social youth training institutions (such as the employment of professional coaches, venue rental, and purchase of training equipment) requires continuous capital investment, and their services need to be purchased by families at their own expense (such as training fees and equipment fees). Per capita disposable income directly reflects the scale of funds that families can freely dispose of; families in economically developed regions are more capable of bearing the “essential costs” of teenagers’ participation in football, thereby promoting the expansion of market demand for social youth training institutions [37,38]. At the same time, in regions with high per capita consumption expenditure, residents have a stronger willingness to consume in sports and leisure, and social capital will actively enter the youth football training field (e.g., establishing branded youth training institutions) to seize business opportunities, forming a positive cycle of “consumption demand → capital investment → growth in the number of institutions”. In addition, social youth training institutions are an important force in the era of professionalization and marketization, and their development is directly linked to regional economic vitality [41]. For example, social youth training institutions in economically developed regions are more likely to obtain corporate sponsorships (such as cooperation with football equipment brands) and can attract high-quality coaching resources (higher-income regions can offer higher salaries), which further strengthens their positive correlation with economic indicators.

Secondly, there is a negative correlation between provincial competitive development indicators and the number of featured schools. Campus football featured schools are government-led carriers for football popularization, and their scale in terms of quantity and the logic behind resource investment are completely different from those of market-oriented entities [25]. They may even show a negative correlation with economic development indicators due to factors such as “policy priority and competition for educational resources”. Featured schools are government-led youth football development programs with a low degree of marketization, and they are not determined by the local economic level [25]. For instance, economically underdeveloped regions may add more characteristic schools due to the policy orientation of “addressing weaknesses”; in contrast, economically developed regions had already completed the layout of characteristic schools in the early stage, resulting in a small number of new additions in the later stage. This “policy compensation effect” leads to a statistically negative correlation where “higher economic level leads to slower growth in the number of characteristic schools”. In addition, the core purpose of featured schools is to enable more students to have access to football and promote football culture, rather than meeting the needs of high-cost elite training. In economically underdeveloped regions, characteristic schools may be the only way for local students to access football, so the policy promotion efforts are stronger, which further intensifies the negative correlation between “economic level and the number of featured schools”.

Finally, there is no significant correlation between provincial economic development indicators and the number of campus football fields, the number of sports schools, or the level of clubs in the three-tier league system. For campus football fields, their construction mainly relies on special funding allocations from the Ministry of Education. For instance, the state may provide additional subsidies for the construction of campus football fields in central and western regions, decoupling the number of such fields from the local economic level. Regarding sports schools, their selection criteria are formulated by the General Administration of Sport of China (e.g., completeness of youth training systems, history of talent cultivation and transfer), which has no connection to the local economic level. For example, sports schools in some economically underdeveloped regions may be included in national talent reserve bases due to their regional advantages in talent resources; in contrast, sports schools in economically developed regions might not be selected due to competition from other sports (e.g., prioritizing the development of basketball or tennis). This results in no correlation between the number of sports schools and economic indicators. As for the level of clubs in the three-tier league system, while there is a certain correlation with economic indicators (e.g., GDP has a positive impact on club level), the economic growth of some regions relies on resource-based industries or heavy industry. Such industries have extremely weak relevance to youth football training and cannot be converted into financial investment or talent reserves for clubs.

In essence, the differentiated correlation between provincial economic development levels and the scale of youth football development stems from differences in the “resource dependence logic” of various development entities. Specifically, social training institutions rely on market consumption and capital investment, which aligns positively with economic indicators; featured schools depend on policy directives and popularization needs, showing no direct correlation or even a negative correlation with economic indicators; campus football fields and sports schools rely on national special policies and functional positioning, being completely independent of local economic levels. This characteristic also confirms the perspective of systems theory—that is, youth football development is an organic system following the chain of “economic input → scale transmission → strength output”. However, different “transmission elements” (social institutions, featured schools, and fields) vary in their sensitivity to “economic input”, ultimately resulting in a differentiated correlation.

### 5.3. Level of clubs in the three-tier league system is positively correlated with the competitive strength of youth football

The results of this study indicate that the level of clubs in the three-tier league system shows a positive correlation with the number of top teams and elite teams, while the number of featured schools exhibits a significant negative correlation with the number of top teams. The core reason for the difference between these two types of correlations lies in the fundamental differences in resource dependence logic, functional positioning, and operational mechanisms among different football development entities.

Firstly, clubs in the three-tier league system (China Super League, China League One, and China League Two) serve as the core carriers for elite youth football development in China [42]. The positive correlation between their level and the number of top teams/elite teams stems from the vertical youth training chain and resource concentration of professional clubs, which is highly consistent with the core viewpoints of the RBV in this study [18,19]. Clubs in the three-tier league system, especially those in the China Super League and China League One, usually have standardized U-series youth teams, equipped with professional coaching teams, scientific training equipment, and competition resources. They are capable of selecting promising young players from the grassroots level and developing them into high-level players through long-term systematic training. The teams composed of these players are inherently qualified to compete for top teams and elite teams.

Secondly, campus football featured schools are government-led carriers for football popularization [25]. The negative correlation between their quantity and the number of top teams mainly stems from the fact that the core purpose of featured schools is to enable more students to get access to football (e.g., offering football interest courses and organizing campus leagues), rather than providing high-intensity elite training. Due to the need to balance students’ academic pressure, featured schools cannot invest a large amount of time in tactical drills and specialized physical training like professional clubs. Moreover, their coaches are mostly school physical education teachers (not professional football coaches), resulting in insufficient scientific rigor and professionalism in training, which makes it difficult to cultivate high-level players.

### 5.4. Level of clubs serves as the core bridge through which the economy influences competitive strength

The results of this study show that the level of clubs in the three-tier league system serves as the core bridge through which the economy influences competitive strength. Specifically, provincial economic development indicators such as GDP, per capita GDP, and per capita disposable income positively affect the level of clubs in the three-tier league system, which in turn positively influences the number of top teams and elite teams.

Firstly, clubs in the three-tier league system have the attribute of resource dependence, and provincial economic development indicators can provide a material foundation for the improvement of their level. Specifically, as professional youth training carriers, clubs in the three-tier league system rely heavily on economic input for their youth team construction, coaching teams, training equipment, and competition resources. GDP, per capita GDP, and per capita disposable income precisely provide resource support for the clubs from different dimensions.

Secondly, clubs in the three-tier league system are the core entities for elite youth football development in China. Their unique vertical youth training chain (selection- cultivation-promotion) is irreplaceable by other football development carriers, which makes them the key hub for converting economic resources into competitive strength. Clubs in the three-tier league system have a standardized youth training system, which can directly convert resources from economic input into players’ abilities. These players are exactly the core force composing top teams and elite teams. In addition, youth training players of clubs in the three-tier league system can directly enter the clubs’ youth teams, and outstanding ones can even be promoted to the first team. This internal promotion channel ensures that high-quality players will not be lost. When GDP, per capita GDP, and per capita disposable income increase the reserve of high-quality players by improving the level of clubs, the youth teams composed of these players inherently have the strength to compete for the top/elite teams in the China Youth Football League (CYFL), and finally realize the positive transmission of “economic indicators → club level → number of top/elite teams”.

At present, the cited document is named plos one revised.docx. The following answer is based solely on this document, and discussions about other documents are ignored. In addition, this study found that in the correlation analysis, the correlation between the level of clubs in the three-tier league system and economic indicators was not significant, but it was significant in the path analysis. The main reason lies in the following aspects. The core of correlation analysis is to calculate the linear correlation coefficient between two variables. It only controls two basic variables, namely total population size and youth population size, and does not consider the multicollinearity within economic indicators. Economic indicators include GDP, per capita GDP, per capita disposable income, and per capita disposable consumption expenditure, and there is a significant correlation among these indicators. Such multicollinearity will lead to the dilution of the independent explanatory power of a single economic indicator, and finally manifest as an insignificant correlation coefficient. By contrast, the goal of path analysis is to verify the chain mechanism among variables. Through the structural equation model, the association of “economic indicators → club level” is placed in the systematic framework of “resource input → scale transmission → strength output”. The statistical test focuses more on “whether the mechanism is valid” rather than the direct association of a single variable. Therefore, it can identify the significant association that is masked in the correlation analysis.

### 5.5. Theoretical contributions and policy recommendations of this study

This study enriches the theoretical research on the relationship between regional economy and specific sub-sectors of the sports industry. With the RBV and the systems theory of youth football development as the core frameworks, this study systematically explores the relationship among the level of provincial economic development, the scale of youth football development, and competitive strength in China. It clarifies the chain structure of the youth football development system, namely “resource input → scale transmission → strength output”, and reveals the differential sensitivity of different “transmission elements” (such as social training institutions, characteristic schools, and football fields) to “economic input”. This finding deepens the theoretical understanding of “how regional economy affects specific sub-sectors of the sports industry” and also provides a theoretical reference for subsequent studies on the relationship between regional economy and the development of other sports programs.

At the policy level, based on the differences in correlations between provincial economic development levels, the scale of youth football development, and competitive strength, this study suggests that the government should formulate targeted policies according to the economic endowments of each province. For provinces with a high total GDP, the focus should be on evaluating the “input-output ratio” of youth football training to avoid inefficient use of resources and guide funds toward core areas such as club construction and coach development. For provinces with a high per capita GDP, social capital should be encouraged to participate in youth football development; through policies like tax incentives and venue support, enterprises can be attracted to invest in social training institutions and sponsor youth football events. For provinces with high residents’ consumption vitality, efforts should focus on fostering market-oriented youth training models. Combined with the results of the mediating effect test, the level of clubs in the three-tier league system is the core carrier through which the economy influences the competitive strength of youth football and thus requires focused efforts. It is recommended that the government work with social forces to support the development of these clubs—this can be achieved through policy preferences (e.g., simplifying event approval procedures, providing training bases) and financial subsidies (e.g., covering youth training costs and coaches’ salaries) to help clubs attract outstanding coaches and update training equipment. Meanwhile, a “linkage mechanism between club youth training and campus football” should be established to encourage clubs to select reserve talents from featured schools, thereby opening up a talent transfer channel connecting the “campus and professional sectors”.

Finally, the study found a negative correlation between the number of campus football featured schools and the number of top-tier and elite teams, reflecting the limitations of featured schools in cultivating elite talents. Therefore, it is suggested to promote in-depth cooperation between featured schools, social brand youth training institutions, and clubs in the three-tier league system: professional coaches should be brought in to participate in the design of campus football courses and training guidance to enhance the professionalism of campus training; the campus football competition system should be improved by linking campus leagues with social competitions and club youth training competitions, providing students with more high-level competitive opportunities; at the same time, a “youth football talent database” should be established to realize the sharing of talent information between campuses and professional institutions. In addition, the assessment criteria for featured schools should be optimized, shifting from “quantity expansion” to “quality improvement”, and indicators such as “talent transfer rate, event participation rate, and students’ football skill compliance rate” should be included in the assessment to avoid the problem of “emphasizing form over effectiveness” in policy implementation.

### 5.6. Limitations of this study

This study reveals the relationship between the economic development level, the scale of youth football development, and football competitive strength. It has positive significance for the formulation of relevant policies. However, this study also has some limitations. Firstly, in measuring the level of economic development, although common indicators such as GDP, per capita GDP, per capita disposable income, and per capita consumption expenditure were selected, these indicators may not fully reflect the complexity and diversity of regional economies. For example, factors such as industrial structure and the sustainability of economic development may also have potential impacts on youth football development, but they were not included in the study. Nor were indicators measuring the economic benefits of football development entities (such as clubs and sports schools) included, while study [43] found a correlation between the balance of payments of football clubs and their competitive performance. Secondly, in terms of measuring the scale of provincial youth football development, it is only reflected through the number of football pitches and the number of youth football organizations. Such quantitative indicators cannot fully reflect the quality of youth football development, and the study has not adopted more precise measurement methods (such as football participation rate, number of professional coaches, youth training duration, etc.) to represent the development scale. Thirdly, the data used in this study are mainly from a specific year and do not fully consider the dynamic changes over time. Both economic development and youth football development are dynamic processes, and the relationships between various indicators may change over time. Conclusions drawn from static data may not accurately reflect long-term trends. In addition, this research design also fails to reveal the causal relationship between variables. For instance, study [44] found that football development can predict regional economic growth. Finally, although a relationship between economic development levels and the scale and competitive strength of youth football development was found, it is not possible to clearly determine that economic factors are the direct cause of changes in football development. There may be other unconsidered factors that simultaneously affect both economic and football development, or there may be complex interactions and feedback mechanisms between the two.

To more deeply and comprehensively explore issues related to youth football development, future research can be expanded in the following directions. Firstly, optimize the indicator system and data collection. When measuring the level of economic development, include more indicators that reflect economic structure and sustainable development to more comprehensively depict the potential impact of regional economic characteristics on youth football development. For assessing the scale of youth football development, in addition to existing quantitative indicators, more data can be obtained through questionnaires and expert evaluations to construct a multi-dimensional comprehensive indicator system. Secondly, conduct dynamic data collection and updating. Establish a long-term tracking data collection mechanism to regularly collect economic data and youth football development data from different years, forming time series data, so that dynamic analysis methods can be used to study the dynamic relationships between variables.

## 6. Conclusion

This study systematically explores the relationship between the level of provincial economic development, the scale of youth football development, and competitive strength in China, and finds that per capita disposable income shows a positive correlation at the marginal significance level with the number of top youth football teams and elite youth football teams; social youth football training institutions exhibit a moderate positive correlation with all economic indicators, reflecting their high dependence on market consumption and social capital investment; campus football featured schools demonstrate a moderate negative correlation with per capita disposable income and per capita consumption expenditure, which is mainly attributed to the policy compensation effect; while indicators such as the number of campus football fields, the level of clubs in the three-tier league system, and the number of sports schools show no significant correlation with economic indicators. The level of clubs in the three-tier league system is the core mediating variable connecting provincial economic development and youth football competitive strength. There is a significant negative correlation between the number of campus football featured schools and the number of top teams, which reflects the limitations of featured schools in cultivating elite talents. In summary, this study clarifies the chain mechanism of “resource input → scale transmission → strength output” in China’s provincial youth football development system, and confirms that the impact of economic development on youth football is not universal but depends on the resource dependence logic of different development entities.

## Supporting information

S1 File Raw dataset used for statistical analysis in this study. (XLSX)
